# Ethical and equity challenges in employment: Perspectives of international nursing graduates

**DOI:** 10.1177/09697330251333397

**Published:** 2025-04-14

**Authors:** Animesh Ghimire, Yunjing Qiu

**Affiliations:** 2541 Monash University; 1994 University of Technology Sydney

**Keywords:** international nursing graduates, employment barriers, systemic discrimination, policy reforms, cultural inclusion

## Abstract

**Background:**

Australia faces a critical shortage of nurses, yet international nursing graduates (INGs) encounter significant barriers to securing employment after graduation. Current policies often prioritize domestic graduates, creating systemic disadvantages for INGs, particularly those on temporary visas. This inequity raises ethical concerns and undermines Australia’s ability to fully utilize its nursing workforce, potentially compromising the quality of healthcare services.

**Aim:**

This study explores the lived experiences of INGs regarding employment challenges in Australia, critically examining the ethical and equity implications of existing practices.

**Research Design:**

A qualitative study employing a combined phenomenological and exploratory approach was conducted. Data were analyzed using a thematic analysis framework.

**Participants and Research Context:**

Twelve international nursing students in their final semester of the Bachelor of Nursing program at two metropolitan universities in Australia participated in semi-structured interviews.

**Ethical Considerations:**

Ethical approval was obtained from the Monash University Human Research Ethics Committee (MUHREC-44400) and the University of Technology Sydney (ETH24-10028). Informed consent was obtained from all participants, who were assured of their right to confidentiality and to withdraw from the study at any time.

**Results:**

Five overarching themes emerged: (1) Economic Disparity and Ethical Considerations, (2) Systemic Discrimination and Inequality, (3) Mental Health and Well-being, (4) Policy and Regulatory Barriers, and (5) Lack of Cultural Inclusion and a Sense of (Un)Belongingness.

**Conclusions:**

The findings highlight urgent ethical concerns and equity challenges that demand comprehensive reforms to create a more inclusive and ethically sound environment for INGs in Australia. These reforms necessitate policy changes to address discriminatory practices and visa restrictions, enhanced institutional support to facilitate INGs’ transition into the workforce, and a commitment to cultural competence and inclusion at all levels of the healthcare system. Addressing these systemic barriers is not only a matter of fairness and justice but is also crucial for ensuring a robust and ethically sustainable healthcare workforce in Australia.

## Introduction

In an increasingly interconnected world, international education, driven by globalization and neoliberal policies, presents a complex interplay of “brain gain” for host countries and “brain drain” for countries of origin.^1–3^ High-income nations like Australia, facing critical nursing shortages,^4,5^ benefit from the skills of international nursing students, who are formally designated as temporary visa holders enrolled in an undergraduate nursing program.^6–8^ While the COVID-19 pandemic disrupted international education,^9^ Australia remains a major destination, with international students injecting an estimated AUD 36.4 billion into the Australian economy.^10–12^ However, this economic contribution, primarily driven by significantly higher tuition fees compared to domestic students,^12,13^ raises ethical questions about the sector’s reliance on these fees. Given the well-documented employment challenges encountered by international nursing graduates (INGs)—defined as individuals who have obtained their nursing qualifications in Australia while on a temporary student visa—it is crucial to examine the systemic barriers and factors that impact their integration into the workforce.^13,14^

## Background

International nursing students occupy a critical position within the Australian healthcare system, representing a vital source of talent to mitigate workforce shortages due to an aging population and growing demand.^15,16^ However, securing a graduate nurse position, a crucial transition from academic training to professional practice, is often uncertain for INGs.^15^ While graduate nurse programs, often based on Benner’s novice-to-expert model,^17^ aim to provide a structured and supportive environment for developing clinical skills and professional identity,^18^ general challenges such as skill development, organizational understanding, and building self-concept^19^ can lead to high turnover if not addressed.^20^ Crucially, visa restrictions, cultural differences, and potential hiring biases compound these challenges for INGs, further magnifying the difficulties faced by this group.

In Australia, the majority of graduate nurse positions are within the public health systems of each state, creating a competitive landscape for aspiring nurses. New South Wales Health (NSW Health), the public health system operating in the state of New South Wales, exemplifies this competitive environment as the largest employer of healthcare workers in the country.^21^ Despite actively recruiting over 3,400 graduate nurses annually, reflecting the high demand for these positions,^22^ the selection process, with its specific eligibility criteria and limited number of positions, presents a significant hurdle for many qualified graduates.^23^

Furthermore, an analysis of the “Graduate Start Recruitment Handbook” from NSW Health reveals a concerning trend: an apparent preference for domestic candidates.^24^ This bias towards international graduates is not unique to NSW Health. A review of recruitment practices across Australia reveals a systemic disadvantage for international nursing graduates. For instance, Victoria Health is part of the state government Department of Health and the second-largest employer of healthcare workers in the state of Victoria.^25^ Previously, it deemed international nursing graduates ineligible for the Postgraduate Medical Council of Victoria’s graduate nurse matching program, effectively excluding them from applying for a graduate nurse position.^26^ This systemic bias, embedded within recruitment practices and policies, presents a formidable barrier for international nursing graduates striving to contribute their skills and expertise to the Australian healthcare system.^27^

The apparent preference for domestic nursing graduates within the Australian healthcare system, as evidenced by the current recruitment practices of NSW Health, raises profound ethical concerns. This bias contradicts the principles of equity and fairness championed by the leading international organization, the International Council of Nurses (ICN). ICN advocates for the ethical treatment of healthcare workers, regardless of nationality, emphasizing the importance of diversity and inclusivity in achieving health equity and mitigating healthcare disparities.^28^ The preferential treatment of domestic graduates, despite the comparable qualifications of their international counterparts, undermines these principles and perpetuates a system that disadvantages a significant portion of the nursing workforce. This practice raises concerns about Australia’s commitment to fostering a truly inclusive and equitable healthcare workforce that reflects the diversity of its population and upholds the ethical principles of fairness and justice.

To bridge the gap between the ethical ideals of global health equity and the lived realities of INGs in Australia, it is essential to center their voices and understand their perspectives on the systemic barriers they encounter. While existing research explores challenges faced by international students generally, including visa restrictions, employment difficulties, and cultural adjustment,^29,30^ there is a critical need for in-depth understanding of the specific experiences of INGs. Previous studies offer insights into job-seeking and employment outcomes, noting visa status, lack of experience, and discrimination,^10,31,32^ but often encompass various disciplines, not fully capturing the unique challenges within nursing. Therefore, this study adopts a qualitative approach to address this gap, prioritizing the in-depth, lived experiences of INGs regarding their employment opportunities and challenges in Australia.

## Aim

This study aims to answer the following question: What are the perspectives of international nursing graduates on their employment opportunities and challenges after graduation in Australia?

## Methods

### Design, population, and setting

This study employed a qualitative research design incorporating both phenomenological and exploratory approaches to delve into the lived experiences and perspectives of international nursing graduates (INGs) regarding their employment opportunities and challenges in Australia post-graduation. The combined phenomenological and exploratory approach was chosen to capture the essence of individual experiences while also allowing for a broader exploration of the contextual factors and systemic issues that shape these experiences. Specifically, this study utilized a descriptive phenomenological approach, focusing on describing the lived experiences of INGs as they are, without attempting to interpret or analyze them within a pre-existing theoretical framework. Phenomenology, in this descriptive form, enables an in-depth understanding of the meaning individuals ascribe to their lived realities,^33^ while the exploratory approach facilitates the identification of patterns, relationships, and potential solutions within a complex phenomenon.^34^

Participants were purposefully selected from two metropolitan universities in Melbourne and Sydney, Australia. These universities were chosen because they have large enrollments of international nursing students and are located in major metropolitan areas with diverse healthcare settings. The timing of the interviews, during the final semester of the student’s final year of the Bachelor of Nursing program, was strategically chosen to capture their perspectives at a critical juncture in their academic and professional journey. This cohort is nearing the completion of their undergraduate studies and actively preparing for their transition to the workforce, making their insights particularly relevant to understanding the challenges and opportunities ahead. This purposive sampling strategy allowed for the selection of participants who could best illuminate the phenomenon under investigation. One-on-one, semi-structured interviews were conducted in a private setting within the university campus, providing a comfortable and confidential environment for participants to share their experiences openly. The semi-structured interview format was chosen for its capacity to elicit rich, in-depth narratives while also allowing for flexibility to explore emerging themes and unexpected insights, fostering a deeper understanding of the participants’ perspectives.

### Participant recruitment

Participants were recruited through a multi-faceted approach designed to reach a broad audience of eligible students. Recruitment materials, including flyers and posters, were strategically placed across prominent locations on both university campuses, such as student notice boards, common areas within the nursing faculty, the university library, and online student forums. Each flyer and poster incorporated a scannable QR code that directed interested individuals to a secure online platform where they could voluntarily register their interest in participating in the study. Eligibility criteria were clearly stated on all recruitment materials and the online registration form. These criteria included: (1) being an international student, defined as a student currently enrolled in the Bachelor of Nursing program on a student visa; and (2) being in the final year and final semester of the program. The online registration form provided a concise overview of the research aims, eligibility criteria, and participant expectations, emphasizing the voluntary nature of participation and the right to withdraw at any stage without penalty. Furthermore, announcements about the study were made during relevant nursing classes and circulated through student email lists, ensuring that potential participants had multiple opportunities to learn about the study and express their interest. Of those who registered interest, all who met the eligibility criteria and provided informed consent after being presented with a detailed information sheet and consent form were included in the study. This information sheet outlined the study’s purpose, procedures, potential risks and benefits, and the participants’ rights, including the right to withdraw at any time. This comprehensive recruitment strategy aimed to ensure a diverse and representative sample of international nursing students while upholding the highest ethical standards of informed consent and voluntary participation.

### Data collection

Data collection occurred between February 2024 and August 2024. The first author (AG), a qualified researcher with extensive experience in conducting qualitative interviews, conducted all face-to-face interviews. To ensure participant comfort and facilitate open sharing, interviews were conducted at a time and location convenient for each participant, with privacy and confidentiality strictly maintained. Each interview lasted between 45 and 75 minutes and was audio-recorded with the participant’s explicit consent.

A semi-structured interview guide, informed by a comprehensive review of relevant literature and the authors’ personal experiences as former international students and migrant nurses. The guide comprised open-ended questions that encouraged participants to elaborate on their experiences, challenges, and perceptions regarding their employment opportunities in Australia post-graduation. This approach allowed for in-depth exploration of individual experiences while also ensuring consistency in the topics covered across interviews. A comprehensive list of the interview questions is provided in Table 1. Following each interview, the first author transcribed the audio recordings verbatim. The transcripts were then reviewed and cross-checked against the audio recordings by the second author (YQ) for quality assurance.

**Table 1.** Interview questions.

Question ID	Interview question
Q1	Can you describe your overall experience as an international nursing student in Australia?
Q2	What specific challenges have you faced in securing employment after graduation?
Q3	How do you perceive the support provided by your university in terms of job preparation and placement?
Q4	In what ways have language and communication barriers affected your job search and professional integration?
Q5	Can you discuss any instances where you felt discriminated against or unfairly treated in the job market?
Q6	How have financial pressures impacted your job search and overall well-being during your studies?
Q7	What has been your experience with visa regulations and work permits in securing employment?
Q8	How accessible have professional networks and mentorship opportunities been for you as an international student?
Q9	How do you feel your cultural background has influenced your job search and integration into the workforce?
Q10	What types of support would you suggest could improve the employment prospects for future international nursing graduates?

### Data analysis

The interview data underwent a rigorous thematic analysis process guided by Braun and Clarke’s,^35^ six-phase framework. This widely recognized framework provides a systematic and flexible approach to identifying, analyzing, and reporting patterns (themes) within qualitative data. In line with this framework, both authors conducted the analysis independently, beginning with a deep immersion in the data through repeated reading and re-reading of the interview transcripts. An inductive approach was adopted, allowing codes and themes to emerge organically from the data rather than being imposed a priori.

The initial coding phase involved open coding, where descriptive codes were assigned to segments of the transcripts that captured key concepts, ideas, and experiences shared by the participants. These codes were then systematically organized into categories based on shared patterns and conceptual similarities. Through a process of constant comparison, where codes and categories were continuously compared and contrasted across transcripts, these categories were further refined and grouped into sub-themes, which were subsequently integrated into overarching themes that captured the essence of the participants’ experiences. Throughout the analysis, the two authors engaged in regular discussions to compare and contrast their coding and interpretations, ensuring consistency and rigor in the analysis process. Any discrepancies were resolved through a collaborative process of revisiting the transcripts and engaging in constructive dialogue until a consensus was reached. This iterative process of independent coding, comparison, and discussion enhanced the trustworthiness and credibility of the analysis.

While data saturation is often considered a key goal in qualitative research, the exploratory nature of this study meant that achieving thematic saturation, where no new themes or codes emerged from the data, was prioritized.^36^ This involved identifying repetitive codes and themes until no new information or relationships emerged from the data.^37^ The richness and depth of the data obtained from the twelve participants indicated that thematic saturation was achieved, as no new codes or themes surfaced in the final stage of data analysis.^38^

The final analysis yielded five central themes, each encompassing several sub-themes and categories, supported by illustrative quotes from the participants. Table 2 provides a detailed overview of the themes, sub-themes, categories, codes, and illustrative quotes that emerged from the data analysis.

**Table 2.** Illustrative quotes, codes, categories, sub-themes, and themes.

Illustrative quotes	Code	Category	Sub-theme	Theme
“We pay so much more than local students, yet we are the last to be considered for jobs.” (P11)	Paying more tuition	High tuition fees	Financial burden	Theme 1: Economic disparity and its ethical implications
“The whole system seems designed to benefit the local students at our expense. […]” (P3)	Prioritization of local candidates	Unjust treatment	Unfair policies	Theme 1: Economic disparity and its ethical implications
“It’s not just about the money […] We’re told we’re welcome here, but then we’re treated like second-class citizens when it comes to jobs.” (P6)	Feeling taken advantage of	Exploitation	Ethical implications	Theme 1: Economic disparity and its ethical implications
“I have seen job postings explicitly stating a preference for citizens or permanent residents […]” (P3)	Preference for locals	Job market discrimination	Biased hiring practices	Theme 2: Systemic discrimination and inequality
“I have seen job postings explicitly stating a preference for citizens or permanent residents.” […] (P3)	Work authorization issues	Visa restrictions	An outsider	Theme 2: Systemic discrimination and inequality
“The constant worry about finding a job affects my mental health. I often feel anxious and stressed, wondering if all my efforts will be in vain.” (P2)	Constant worry	Job search anxiety	Anxiety and stress	Theme 3: Mental health and well-being
“I’ve started to doubt my abilities and question my decision to study here.” (P1)	Questioning abilities	Self-doubt and decision regret	Loss of confidence	Theme 3: Mental health and well-being
“I am on a student visa with restricted work hours while applying for graduate nurse positions that require us to work full-time […]” (P3)	Complicated visa process	Stringent visa conditions	Visa requirements	Theme 4: Policy and regulatory barriers
“My sister graduated with a bachelor of nursing and […]. However, due to a delay in getting her post-graduate work visa, [..].” (P11)	Employer reluctance	Sponsorship challenges	Difficulties with sponsorship	Theme 4: Policy and regulatory barriers
“Even if I do get a graduate work visa, it is only valid for 2 years, and within that time, I either will have to get a permanent resident or find an employer to sponsor me or leave the country [….]” (P9)	Short-term visa validity	Temporary work permits	Visa-related instability	Theme 4: Policy and regulatory barriers
“I find it hard to connect with mentors and preceptors who do not understand my background and the specific challenges I face as an international student […].” (P7)	Lack of mentor connections	Belongingness	Cultural barriers	Theme 5: Lack of cultural inclusion and feeling of (Un)belongingness
“Cultural differences can be a barrier. I Sometimes feel out of place or misunderstood […].” (P6)	Feeling out of place	Exclusion	Social and professional isolation	Theme 5: Lack of cultural inclusion and feeling of (Un)belongingness
“There is a lack of diversity in leadership roles within healthcare institutions […].” (P1)	Lack of diversity in leadership	Leadership representation	Underrepresentation in leadership	Theme 5: Lack of cultural inclusion and feeling of (Un)belongingness

### Rigor and reflexivity

To ensure the rigor and trustworthiness of this qualitative study, we adhered to Lincoln and Guba’s,^39^ four criteria for evaluating qualitative research: credibility, transferability, dependability, and confirmability.

Credibility, which refers to the accuracy and truthfulness of the findings, was established through member checking. Prior to the data analysis, the transcribed raw data was sent to the participants for verification, allowing them to add, remove, or modify any comments to ensure the accurate representation of their perspectives. Transferability, the extent to which the findings can be applied to other contexts or settings, was enhanced by providing rich, detailed descriptions of the study context, participant demographics, and research process. This rich contextual information allows readers to assess the applicability of the findings to their own situations. Dependability, the consistency of the findings over time and across researchers, was addressed by meticulously documenting the research process, including the development of the interview guide, data collection procedures, and analysis techniques. This detailed documentation allows for the study to be audited and potentially replicated by other researchers. Confirmability, the degree to which the findings are grounded in the data and not influenced by researcher bias, was promoted through several strategies. Both authors conducted the data analysis independently, followed by a collaborative process of comparing and contrasting their interpretations to reach a consensus. This ensured that the findings were rooted in the data and not solely reflective of individual perspectives.

Furthermore, the research team acknowledges the importance of reflexivity in qualitative research and the imperative of critically examining our own positionality and potential biases. The research team, comprising nursing academics with diverse backgrounds, including experiences as international students and migrant nurses, actively engaged in critical self-reflection throughout the research process. We recognize that our personal experiences may have influenced our research questions, data collection, analysis, and interpretation. To mitigate this potential bias, we maintained reflective journals to document our thoughts, assumptions, and potential biases throughout the study. Regular discussions within the research team and with external colleagues further facilitated critical examination of our interpretations and ensured that our personal experiences did not unduly influence the research findings. While acknowledging that our backgrounds may have predisposed us to certain interpretations, we actively sought to challenge our assumptions and remain open to alternative perspectives throughout the research process.

### Ethical considerations

Ethical approval was obtained from the Monash University Human Research Ethics Committee (MUHREC-44400) and the University of Technology Sydney (ETH24-10028). We sought informed consent from each participant, both verbally and through written information, and they were assured of their right to withdraw from the study at any time without any consequences.

## Results

### Participants’ characteristics

The demographic characteristics of the study participants are presented in Table 3. Of the initial 14 individuals who registered interest, 12 proceeded to provide informed consent and participated in the study. All participants had been residing in Australia for nearly 3 years, which is the typical duration of the Bachelor of Nursing program. The sample included twelve international nursing students in their final year and semester of a Bachelor of Nursing program at metropolitan universities in Melbourne and Sydney, Australia. Their ages ranged from 22 to 27 years, with a majority (8 out of 12) identifying as female. Two male participants were recruited from each participating university from Sydney and Melbourne. The participants represented five key source countries for international nursing students in Australia: Nepal (*n* = 3), India (*n* = 3), The Philippines (*n* = 1), Indonesia (*n* = 2), and China (*n* = 3). This distribution aligns with national data on international student enrollment, where China, India, and Nepal consistently rank among the top countries of origin for international students in Australia.^40^

**Table 3.** Participant demographics.

Participant ID	Country of origin	Gender	Year of study	Visa status	Nursing as a first choice?
P1	Nepal	Female	3rd year, 2nd semester	Student visa	Yes
P2	Nepal	Male	3rd year, 2nd semester	Student visa	No
P3	Nepal	Female	3rd year, 2nd semester	Student visa	Yes
P4	The Philippines	Female	3rd year, 2nd semester	Student visa	Yes
P5	India	Male	3rd year, 2nd semester	Student visa	No
P6	India	Female	3rd year, 2nd semester	Student visa	Yes
P7	India	Female	3rd year, 2nd semester	Student visa	Yes
P8	Indonesia	Female	3rd year, 2nd semester	Student visa	Yes
P9	Indonesia	Male	3rd year, 2nd semester	Student visa	No
P10	China	Female	3rd year, 2nd semester	Student visa	Yes
P11	China	Female	3rd year, 2nd semester	Student visa	Yes
P12	China	Male	3rd year, 2nd semester	Student visa	No

The sample also reflects the gender distribution typically observed in the nursing workforce, where females are significantly overrepresented. In 2024, 87.97% of registered nurses in Australia identified as female, compared to 12.01% identifying as male.^41^ Interestingly, all four male participants in this study indicated that nursing was not their first-choice career path. This raises important questions about the persistent underrepresentation of males in nursing and the ongoing perception of nursing as a feminized profession, issues that warrant further investigation.

## Findings

### Theme 1: Economic disparity and ethical implications

The financial burden shouldered by international students, who pay significantly higher tuition fees than their domestic counterparts, was a recurring motif in their narratives. This economic disparity formed the foundation for significant ethical concerns, particularly regarding fairness, justice, and the perceived exploitation of international students.^42^ The juxtaposition of this financial investment with the limited employment opportunities postgraduation, primarily due to policies favoring local candidates, fueled feelings of frustration, disillusionment, and a sense that their contributions were not valued.

“We pay so much more than local students, yet we are the last to be considered for jobs. It feels like we're only here to fill a quota and fund the university.” (P11)

“My family and I invested a lot of money into my education here, hoping to gain valuable work experience with a full-time job, but the reality is that my chances of getting a job are slim due to my visa status. It's disheartening.” (P12)

“The whole system seems designed to benefit the local students at our expense. We pay more and get less in return—less job security, support, and fewer opportunities.” (P3)

“It’s not just about the money; it’s about feeling like we’re being taken advantage of. We're told we’re welcome here, but then we're treated like second-class citizens when it comes to jobs.” (P6)

These expressions expose the economic disparity and, crucially, the ethical concerns that arise directly from this disparity. The participants’ experiences highlight not just the financial burden but also the ethical implications of a system perceived as unjust, exploitative, and failing to provide equitable opportunities.

### Theme 2: Systemic discrimination and inequality

The narratives of the INGs illuminate a deeply entrenched system of discrimination and inequality, extending beyond isolated instances of bias to permeate institutional practices and policies. Their experiences paint a picture of exclusion and unequal access to opportunities, hindering their professional integration and contributing to feelings of marginalization.

“I have seen job postings explicitly stating a preference for citizens or permanent residents. It is disheartening to know that my chances are slim despite being equally qualified because of my visa status. While applying for a new graduate program, it states, ‘ensure you hold the correct visa which provides the Right to Work in Australia’, but during the time of application, I am on a student visa that has work restrictions of 48 hours a fortnight, but the graduate program is full-time. I am worried that my visa process will hold me down even if I get into the program.” (P3)

“Even during clinical placements, I felt like I was treated differently. My supervisors were more critical of my work compared to my local classmates, and I had to work twice as hard to prove myself.” (P7)

“There is a lack of support from the university when it comes to job placements and career advice. Local students have better access to resources and networks, while we are left to fend for ourselves.” (P9)

These testimonies underscore the systemic nature of the discrimination faced by INGs, manifesting overtly and subtly across various domains of their academic and professional journey.

### Theme 3: Mental health and well-being

The precarious nature of their employment prospects takes a toll on the mental health and well-being of INGs. The constant anxiety surrounding job security and the fear of returning home empty-handed after a significant financial and emotional investment creates a pervasive sense of uncertainty and vulnerability.

“The constant worry about finding a job affects my mental health. I often feel anxious and stressed, wondering if all my efforts will be in vain.” (P2)

“I've started to doubt my abilities and question my decision to study here. The stress of not knowing if I'll find a job makes it hard to focus on my studies and enjoy my time.” (P1)

“It is not just about the job itself but the fear of having to leave Australia with no relevant experience.” (P7)

The cumulative impact of these stressors can be profound. They can create a sense of isolation and alienation, hindering their ability to fully integrate into their communities and enjoy their time in Australia.

### Theme 4: Policy and regulatory barriers

The labyrinthine visa regulations and the arduous process of obtaining work authorization in Australia pose formidable obstacles for INGs seeking employment. The stringent requirements and inherent uncertainties associated with temporary visas make many employers reluctant to hire international graduates, resulting in a significant underutilization of this valuable talent pool.

“I am on a student visa with restricted work hours while applying for graduate nurse positions that require us to work full-time. Rather than focusing and asking questions on what areas we might prefer to work in or regarding our interest/choice of specialty, the prospective employer's first question is, “When will you get your post-study work visa?” (P3)

“My sister graduated with a bachelor of nursing and was offered a full-time position in an aged care facility. However, she missed the cut-off date to start her position due to a delay in getting her post-graduate work visa.” (P11)

“Even if I do get a graduate work visa, it is only valid for two years, and within that time, I either will have to get a permanent resident or find an employer to sponsor me or leave the country [….]” (P9)

These voices underscore the precariousness of the INGs’ situation, highlighting the policy and regulatory barriers that create a sense of constant insecurity and limit their ability to plan for their future.

### Theme 5: Lack of cultural inclusion and feeling of (un) belongingness

The INGs’ narratives reveal that the degree of cultural inclusion within healthcare institutions significantly shapes their professional experiences and opportunities. A lack of understanding and recognition of their unique cultural backgrounds and challenges can lead to feelings of isolation and hinder their ability to integrate fully into the workplace.

“I find it hard to connect with mentors and preceptors who do not understand my background and the specific challenges I face as an international student. In our debriefing session with the preceptor, we discussed clinical concepts and the patients we cared for during the shift […]. When the preceptor asks us questions, most of the time, local students are quick to respond […]. Sometimes, I am not up to date with the course materials, and the preceptor tells us (by comparing us with the local students) that they are not working, so why are you? I still have to work to pay my bills even though I work full-time hours doing my placement. People here do not understand what we have to go through […]” (P7)

“Cultural differences can be a barrier. I sometimes feel out of place or misunderstood in professional settings, which makes it harder to form meaningful connections with colleagues and supervisors.” (P6)

“There is a lack of diversity in leadership roles within healthcare institutions, which makes it difficult for international students like me to see themselves represented and feel like they belong.” (P1)

The lack of culturally sensitive mentorship, the feeling of being misunderstood or out of place, and the underrepresentation of diverse leaders all contribute to a sense of alienation and hinder their professional integration.

## Discussion

International students and graduates face barriers to employment, including discrimination against temporary visa holders, limited local networks and work experience, and concerns about English language proficiency.^32,43,44^ This study expands on this body of knowledge by specifically examining the experiences of international nursing graduates (INGs) in Australia, a country with a significant nursing shortage,^4^ yet failing to fully integrate these skilled graduates into its healthcare workforce. This paradox, where a country actively recruits internationally qualified nurses for a field experiencing a significant workforce deficit^45^ yet fails to fully integrate these graduates who have studied and invested their time and resources in Australia, creates a stark contradiction between Australia’s stated commitment to attracting international students^46^ and the reality faced by INGs.

Our findings reveal that visa restrictions play a crucial role in this disparity. Many INGs, even after completing their undergraduate studies, struggle to secure employment due to the limited duration of their graduate visas.^32^ Employers often seek candidates with a minimum of 2 years of remaining visa validity,^32^ a requirement that many INGs cannot meet, especially if their job search extends for several months or a year after graduation.^32^ Current Australian policy offers limited options for graduates to extend or renew their visas, further exacerbating this challenge.^32^ This systemic failure to assimilate INGs, representing a valuable potential solution to the nursing shortage, raises significant ethical, economic, and healthcare workforce concerns. By hindering the professional integration of INGs, Australia not only undermines its own efforts to address critical workforce needs but also perpetuates a system that can be perceived as exploitative and unjust. The term “cash cows” frequently used in Australian media to describe international students,^47^ further illustrates the problematic perception of these students as primarily a source of revenue rather than valuable contributors to the workforce.

Despite the global nature of this issue, national employment policies prioritize local workforce needs, creating systemic barriers for international graduates. The exclusion of temporary residents from unemployment statistics further perpetuates this bias, favoring domestic candidates even in sectors with critical shortages like nursing. While prioritizing domestic students might be understandable from a nationalistic perspective, it raises questions about fairness and equity in the context of a globalized education system. The disproportionate financial burden placed on international students, combined with limited access to employment opportunities, creates a system that can be perceived as exploitative, undermining the principles of social justice and global citizenship.^48^

The pervasive experiences of systemic discrimination and inequality recounted by the participants in this study underscore the formidable challenges INGs face in their pursuit of professional integration in Australia. These findings resonate with the broader literature on international students, which consistently highlights the presence of biases and discriminatory practices that limit their career prospects and create a sense of exclusion.^49–51^ The lack of institutional accountability for supporting the workforce integration of international students further exacerbates this issue.^52^

This systemic discrimination, deeply ingrained within institutional cultures and practices, often manifests in subtle and insidious ways. While policies promoting diversity may exist on paper, their effectiveness is often undermined by the persistence of unconscious biases and a preference for cultural and professional familiarity. This is particularly evident in the Australian healthcare sector, where, despite a growing awareness of the need for cultural diversity, international nurses often report feeling undervalued and excluded.^45^ Internationally qualified nurses in Australia experienced difficulties gaining recognition for their overseas qualifications and faced challenges in navigating the cultural nuances of the Australian healthcare workplace.^45,53^

A multi-pronged approach is needed to dismantle these entrenched barriers, encompassing both top-down and bottom-up strategies. While top-down approaches focus on policy changes and institutional reforms, such as mandatory cultural competency training^54,55^ and removing priority selection based on visa status,^24^ bottom-up strategies emphasize grassroots initiatives and individual empowerment. This could include establishing peer support networks for INGs, creating mentorship programs that connect INGs with experienced nurses from diverse backgrounds, and providing opportunities for INGs to share their cultural knowledge and expertise with their colleagues. By combining these approaches, Australia can create a more inclusive and supportive environment for INGs, allowing them to fully contribute their skills and perspectives to the healthcare system.

A central finding of this study is the impact of employment uncertainties, systemic discrimination, and limited institutional support on the mental health and well-being of international nursing students. The unique challenges faced by these students, including financial pressures, academic demands, and the ongoing process of cultural adjustment, create fertile ground for anxiety and stress.^56,57^ These stressors are further amplified in demanding fields such as nursing, where the pressure to perform academically and professionally can be overwhelming for graduate nurses.^58,59^ Furthermore, the discourse surrounding “work readiness” among graduate nurses frequently emerges within scholarly literature, underscoring its importance as a critical topic in the field.^60–62^ This discussion often highlights the need for comprehensive preparation to ensure that new graduates are effectively equipped to meet the challenges posed by the healthcare environment.

This study highlights that work readiness for INGs is not solely dependent on factors such as the preceptor-student relationship,^63^ the quality of education provided by higher education institutions,^64^ or the support offered by healthcare facilities.^61^ It is also significantly influenced by the student’s individual circumstances, particularly their visa status. The precariousness of temporary visas, with their inherent restrictions and uncertainties,^65,66^ creates an additional layer of complexity for INGs as they strive to meet the expectations of work readiness. This underscores the need for healthcare and higher education organizations to recognize the unique challenges faced by INGs and provide tailored guidance and support to enhance their work readiness and facilitate a smoother transition into the workforce.

Furthermore, the mental health challenges encountered by INGs can be attributed to a complex interplay of factors. Financial constraints, often exacerbated by their ineligibility for social benefits as temporary visa holders^67^ and the high cost of international student tuition contributes to a sense of vulnerability and anxiety.^68^ The uncertainty of securing employment due to preferential hiring practices further compounds these financial pressures, creating a constant sense of instability.^69,70^ Moreover, the experience of discrimination and exclusion, both within academic and professional settings, can erode their self-esteem and sense of belonging.^71,72^ The pressure of acculturation,^57^ navigating new social norms,^73^ and professional expectations,^74^ adds another layer of complexity to their already challenging circumstances.

The restrictive and often unpredictable nature of visa regulations, particularly during the transition from a student visa to a graduate visa,^32^ further contributes to the mental health burden faced by INGs. The constant need to navigate complex bureaucratic processes and the looming possibility of visa expiration create a sense of insecurity,^32^ hindering their ability to fully focus on their studies and professional development. The stark contrast between the opportunities readily available to domestic peers and the limited prospects faced by INGs further exacerbates their sense of vulnerability and marginalization.^75,76^

The plight of “left-out” international students, who feel betrayed by a system that entices them with promises of opportunity but fails to provide adequate support for their integration, resonates deeply with the findings of this study.^77^ The ethical implications of this situation are profound, raising questions about the responsibility of governments and educational institutions to ensure that international students are not merely seen as a source of revenue but as valuable contributors to society. As expressed by the participants, the desire for a fair and equal opportunity to compete in the job market underscores the fundamental principles of justice and equity that should underpin any immigration and education policy.

The lack of cultural inclusion experienced by INGs further compounds their challenges. The subtle and often unintentional biases that permeate healthcare institutions can create a sense of alienation and hinder the professional development of these graduates.^78,79^ As one participant highlighted, the comparison between international and domestic students regarding their need to work during clinical placements exemplifies the lack of understanding and sensitivity towards the unique circumstances INGs face. The research by Patel et al.^80^ and Sakiz & Jencius^81^ emphasizes the importance of cultural competence and inclusivity in both policy and practice to create a supportive environment for international students. The findings of Gonzales^82^ further underscore the detrimental impact of microaggressions on the sense of belongingness among international students. The cumulative effect of these challenges creates a sense of ambiguity for INGs, hindering their ability to fully realize their potential and contribute to the Australian healthcare system.

## Limitations and future research

This study, while providing valuable insights into the experiences of INGs in Australia, is subject to certain limitations. Firstly, the sample size, while sufficient for achieving thematic saturation within the chosen qualitative approach, limits the generalizability of the findings to the broader population of INGs across Australia. The study was conducted at two metropolitan universities in Sydney and Melbourne; therefore, the findings may not fully reflect the experiences of INGs in regional or rural settings or those studying at different types of institutions. Second, the study focused specifically on INGs in their final year of study. Future research could explore the experiences of INGs at different stages of their career trajectory, including those who have been in the workforce for several years, to gain a more comprehensive understanding of long-term integration challenges and successes. This could also explore, through longitudinal design, the longer-term effects of employment challenges identified in this study. Finally, this study focused on the Australian context. Future research could conduct comparative studies across different countries with significant populations of international nursing students to identify common challenges and best practices for supporting their integration into the healthcare workforce. Cross-national comparisons would provide a broader perspective and inform policy development at an international level. Further studies could investigate specific institutional policies and practices that either facilitate or hinder the employment of INGs.

## Recommendation and conclusions

To address the multifaceted challenges faced by INGs in securing post-graduation employment, a concerted effort is required from policymakers, educational institutions, and healthcare providers. The following recommendations, grounded in the study’s findings, offer a roadmap for fostering a more equitable and inclusive environment for INGs in Australia.

First, it is crucial to ensure recruitment equity by mandating the removal of any preferential selection practices based on visa status. This will ensure that INGs have equal opportunities to compete for positions based on their merit and qualifications rather than being disadvantaged by their nationality. Second, educational institutions and healthcare providers should establish targeted career services tailored to the unique needs of INGs. These services should offer support with resume writing, interview skills, and navigating the complexities of the Australian job market, equipping INGs with the tools and knowledge necessary to succeed in their job search. Third, promoting diversity in leadership positions within healthcare institutions is essential to create visible role models for INGs and foster a sense of belonging. Seeing individuals from diverse backgrounds in leadership roles can inspire and motivate INGs, demonstrating that career progression is possible regardless of their nationality. Finally, implementing regular feedback mechanisms to capture INGs’ experiences and challenges is crucial for continuous improvement. This feedback can inform policy changes, refine support services, and create a more responsive and inclusive environment for INGs.

This study has illuminated the complex landscape of systemic challenges faced by INGs in Australia, hindering their professional integration and well-being. The economic disparities, discriminatory practices, policy barriers, and lack of cultural inclusion they encounter create a formidable obstacle course, undermining their aspirations and contributions to the healthcare sector. However, these challenges also present a compelling opportunity for transformative change. By embracing evidence-based policy reforms, fostering institutional support, and prioritizing cultural competence and inclusion, Australia can harness the full potential of its diverse nursing workforce. This not only promises to alleviate nursing shortages but also to enrich the cultural fabric of the healthcare system, ultimately leading to improved patient care and outcomes. The findings of this study serve as a call to action, urging stakeholders to dismantle the systemic barriers that perpetuate inequity and create a healthcare system that truly reflects the values of diversity, equity, and justice. The question remains: Will Australia rise to the challenge and embrace the opportunity to create a truly inclusive and equitable healthcare workforce?

## Data Availability

The data that support the findings of this study are available on request from the corresponding author. The data is not publicly available due to privacy or ethical restrictions.*
